# Optimization of the Consolidation Parameters for Enhanced Thermoelectric Properties of Gr-Bi_2_Te_2.55_Se_0.45_ Nanocomposites

**DOI:** 10.3390/nano14030260

**Published:** 2024-01-25

**Authors:** Farah El-Makaty, Abdel Magid Hamouda, Anas Abutaha, Khaled Youssef

**Affiliations:** 1 Mechanical and Industrial Engineering Department, Qatar University, Doha 2713, Qatar; fe1206656@qu.edu.qa (F.E.-M.); hamouda@qu.edu.qa (A.M.H.); 2 HBKU Core Labs, Hamad Bin Khalifa University, Doha 34110, Qatar; aabutaha@hbku.edu.qa; 3 Materials Science and Technology Graduate Program, Department of Physics and Materials Science, Qatar University, Doha 2713, Qatar

**Keywords:** thermoelectric, n type, mechanical milling, hot pressing, bismuth telluride, nanocomposite

## Abstract

Hot pressing represents a promising consolidation technique for ball-milled bismuth telluride alloys, yet deep investigations are needed to understand its effect on the thermoelectric properties. This paper studies the effect of hot-pressing parameters (temperature and pressure) on the thermoelectric properties of the n-type Gr-Bi_2_Te_2.55_Se_0.45_ nanocomposite. Ultra-high pressure, up to 1.5 GPa, is considered for the first time for consolidating Bi_2_(Te,Se)_3_ alloys. Results from this study show that increasing the temperature leads to changes in chemical composition and causes noticeable grain growth. On the contrary, increasing pressure mainly causes improvements in densification. Overall, increments in these two parameters improve the ZT values, with the temperature parameter having a higher influence. The highest ZT of 0.69 at 160 °C was obtained for the sample hot-pressed at 350 °C and 1 GPa for 5 min, which is indeed an excellent and competitive value when compared with results reported for this n-type Bi_2_Te_2.55_Se_0.45_ composition.

## 1. Introduction

Thermoelectric (TE) materials offer direct and reversible conversion of waste heat to electricity, representing an optimistic green energy source candidate with great potential to aid in overcoming pollution problems once effectively produced [1]. Unlike solar energy, TE devices are less expensive, sunlight-independent, and produce stable voltage over time [2]. Nonetheless, the usage of TE materials in devices has been continually limited by their conversion efficiencies, which mainly depend on the figure of merit (ZT) calculated as

(1) $$ZT=\frac{S^{2}\sigma }{k}\;T$$

where *S* is the Seebeck coefficient, *σ* is the electrical conductivity, *T* is the absolute temperature, and *κ* is the total thermal conductivity [3]. A combination of high-power factor (S^2^σ) and low *κ* is desired to achieve high performance in a TE device. Thus far, the most successful commercially accessible TE materials for low-heat applications are Bi_2_Te_3_ systems [4]. However, the low ZT values (less than 3) only limit their usage for niche applications [1]. 

High performance of both p- and n-type TE materials is necessary to maximize device efficiency [5]. P-type (Bi,Sb)_2_Te_3_ materials have shown significant progress in TE performance. For example, Yang et al. [6] prepared a p-type Bi_0.5_Sb_1.5_Te_3_/boron composite with an ultra-high ZT of 1.6 at 100 °C. Nevertheless, ZT values greater than one have seldom been reported for n-type Bi_2_(Te,Se)_3_ due to their strong anisotropy of charge carrier transport properties. In the past years, various approaches have been implemented to enhance the efficiency of n-type Bi_2_(Te,Se)_3_ alloys, mainly focusing on texturing [7,8], nanoprecipitates [9,10], nanocompositing [11,12] and nanostructuring [11]. Even though texturing is beneficial in raising the ZT of n-type alloys, its significant drawbacks include complexity, high production costs, and poor mechanical performance [13,14]. Hence, the current research primarily focuses on nanostructuring and nanocompositing through powder metallurgy to enhance the ZT of n-type alloys [15]. Dense grain boundaries resulting from nanostructuring and the newly introduced interfaces from nanocompositing effectively lower the lattice thermal conductivity while preserving high electrical conductivity values [5,9,10,11,16]. 

A recent review paper by El-Makaty et al. [1] showed that using 2D nanofillers, such as graphene, in Bi_2_(Te,Se)_3_ nanocomposite results in the highest thermoelectric improvements compared to 1D and 0D nanofillers. The reasons for better improvements in 2D nanofillers include (i) reduced thermal conductivity since scattering of phonons happening at a single nanosheet interface occurs in two dimensions, and (ii) improved electrical conductivity through tunneling behavior is obtained as carriers are allowed to move in two directions. However, researchers reported that using graphene in high amounts (e.g., 3–10 wt.%) may result in an undesired reduction in thermoelectric properties [17]. Hence, optimization of the nanofiller concentration is needed to avoid agglomeration of graphene nanosheets and obtain the high aspect ratio needed for tunneling to take place. 

Out of all techniques, ball milling represents a simple, fast, and economical way to produce nanomaterials. The powders produced are reported to have excellent homogeneity [18], and the method has been broadly utilized to fabricate Bi_2_(Te,Se)_3_ alloys as TE materials [19,20]. Unlike other complex and expensive methods [2,21], ball milling does not produce chemical waste. The main obstacle in ball milling is the need to consolidate the powders produced before employing them in the device. The most common consolidation techniques include hot pressing (HP), spark plasma sintering (SPS), microwave sintering, and hot isostatic pressing (HIP) [22,23]. However, some difficulties may arise during these consolidation methods. For example, the resistive heating used in the SPS and HIP results in modifications to the inherent nanoparticles’ properties as well as noticeable grain growth [24]. In microwave sintering, getting complete control of the applied temperature is challenging, which is greatly influenced by the placement of the sample inside the furnace. Since the heat is generated internally within the sample, the actual temperature is always higher than the measured one [25]. On the other hand, the HP process can provide a more controlled environment for consolidating the ball-milled powders. Nevertheless, optimizing HP parameters, such as temperature, holding time, and pressure, is a must to retain the nano-features and design superior TE properties during the consolidation stage.

Eum et al. [26] reported that Bi_2_Te_2.55_Se_0.45_ is the optimized composition of n-type Bi_2_(Te,Se)_3_ prepared by ball milling and hot pressing techniques that provide superior thermoelectric properties. Moreover, an optimized graphene content of 0.05 wt.% added at the last 10 min of mechanical milling provides a uniform distribution of graphene nanosheets, resulting in maximized thermoelectric performance [11]. In the present investigation, efforts are made to optimize and understand the effect of the hot-pressing conditions to maximize the thermoelectric properties of an n-type Gr/Bi_2_Te_2.55_Se_0.45_ alloy prepared by nanocompositing (with 2D graphene) and nanostructuring (ball milling) methods. The main focus of this work is to systematically choose the best operating temperature and pressure conditions to improve the ZT of the prepared alloy. 

## 2. Materials and Methods

### 2.1. Materials, Synthesis, and HP Optimization of Ball-Milled Gr-Bi_2_Te_2.55_Se_0.45_

Bismuth (99.99%, Sigma-Aldrich, Taufkirchen, Germany), tellurium (99.997%, Sigma-Aldrich), and selenium (99.5%, Alfa Aesar, Haverhill, MA, USA) elemental powders were used to prepare the Bi_2_Te_2.55_Se_0.45_ alloy. The powders were loaded under an inert atmosphere and milled with a ball-to-material weight ratio of 7:1 for 20 h using an SPEX milling device (8000M, SPEX SamplePrep, Metuchen, NJ, USA). In the last ten minutes of mechanical milling, the vial was opened under an argon atmosphere, and 0.05 wt.% of graphene nanoplatelets (Sigma-Aldrich) were added to the alloy, and the milling was resumed. The produced nanocomposite alloy (0.05 wt% Gr-Bi_2_Te_2.55_Se_0.45_) was then consolidated at different conditions (stated in Table 1) as follows: An 18 mm tungsten carbide die is thoroughly sprayed with a conductive graphite-based lacquer (CAMOLIN, Eichenbach, Germany) and allowed to dry. The milled powder is loaded into the die under an argon atmosphere and inserted into an electric heater (TEMPCO MI-Plus, 600W-240V, Wood Dale, IL, USA). A hollow copper cylinder is used between the heater and the die to distribute the heat more uniformly. A thermocouple (TEMPCO MI-Plus, type K-G, USA) is introduced through the heater and the copper cylinder to the die’s outer surface, and the temperature is contentiously measured. The heater arrangement is placed in a hydraulic press (HP50 G3, COMPAC, Juelsminde, Denmark) and adjusted using stainless steel plates. Next, the chamber containing the setup is evacuated three times (RV8, EDWARDS, Stockholm, Sweden) and refilled with pure argon (Ar ≥ 99.999%). A power supply (720W, KEITHLEY, Solon, OH, USA) is used to heat the sample at a constant heating rate of 25 °C/min until the desired temperature is reached. The pressure is then applied and held for 5 min. Once finished, the pressure is released, and the 18 mm sample with a thickness ranging from 2 to 3 mm is allowed to cool inside the chamber, whose base contains water circulating through a chiller (JSR, Chungchungnam-Do, Korea).

### 2.2. Characterization

Phase identification of the hot-pressed Gr-Bi_2_Te_2.55_Se_0.45_ samples was investigated by X-ray diffraction (XRD: EMPYREAN, Malvern PANalytical, Spectris plc, Worcestershire, United Kingdom) under air using a radiation source of Cu/Kα (λ = 1.54 Å). TEM (FEI Titan™ 60–300 TEM, Hillsboro, OR, USA) was carried out to examine the nanostructure of the samples. TEM samples were prepared using a focused ion beam (FEI Helios NanoLab™ G4 FIB/SEM, Hillsboro, OR, USA) dual system. An optical microscope (BX53M OLYMPUS, Tokyo, Japan) was used to obtain surface images. The density of the prepared discs was measured based on Archimedes’ principle using the Sartorius density determination kit (Sartorius YDK03, Göttingen, Germany). The density determination kit was set up using distilled water, and the water’s temperature was monitored during the measurements. Hall voltage was measured using the Lakeshore 8400 device, where the magnetic field was swept to 1 T with a step of 0.2 T. The carrier concentration was calculated from the slope of the Hall voltage as a function of the magnetic field.

### 2.3. Thermoelectric Properties Measurements

The electrical conductivity and the Seebeck coefficient were measured simultaneously for a 25 to 300 °C temperature range under a UHP argon atmosphere using the SBA 485 Nemesis–NETZSCH device (Selb, Germany). The thermal conductivity was measured using a Trident C-Therm device (MTPS Guard Ring Technology, C-Therm, New Brunswick, Canada) with a thermal joint compound (type 120 silicone) as a contact agent. The measurements were performed in a temperature range of 25 to 150 °C. The measurements were repeated five times for each sample at each temperature, and the error bar was less than 9%.

## 3. Results and Discussion

**Figure 1:**
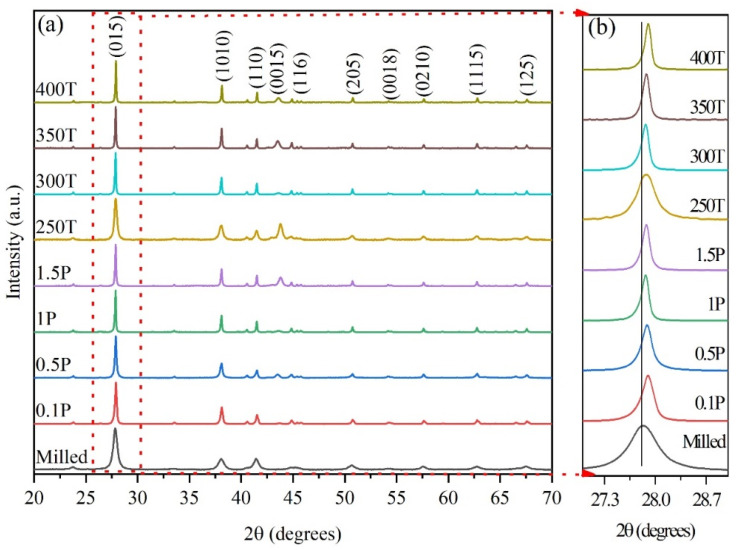
(**a**) XRD patterns of HP Gr-Bi_2_Te_2.55_Se_0.45_ pellets at different conditions. (**b**) enlarged (015) peak of the same samples.

The XRD patterns of the as-milled powder and HP pellets are shown in Figure 1a. All diffraction peaks can be indexed to the rhombohedral lattice (R-3m phase) and are consistent with the standard card number 98-024-7619 for n-type Bi_2_Te_2_Se_1_ [27]. No graphene peaks were present in the diffraction patterns since it was only added in small amounts (0.05 wt.%) [11]. XRD peak broadening is observed for the as-milled sample, indicating high levels of lattice strain and grain refinement due to the severe plastic deformation during mechanical alloying. The XRD peaks of the HP pellets are narrower compared to those of the milled powder, which could be attributed to a reduced level of lattice strain and grain growth after hot pressing [28]. Moreover, increasing the pressing temperature from 250 °C to 400 °C caused a shift in the peaks to higher angles, as seen in Figure 1b. This shift can be explained by the evaporation of constituents, mainly Se and Te, since they have a lower energy of evaporation (37.70 and 52.55 kJ/mol, respectively) compared to Bi (104.80 kJ/mol) [29]. On the other hand, based on the enlarged XRD peaks in Figure 1b, varying pressure (in samples 0.1 P, 0.5 P, 1 P, and 1.5 P) does not seem to affect the crystal structure. 

**Figure 2:**
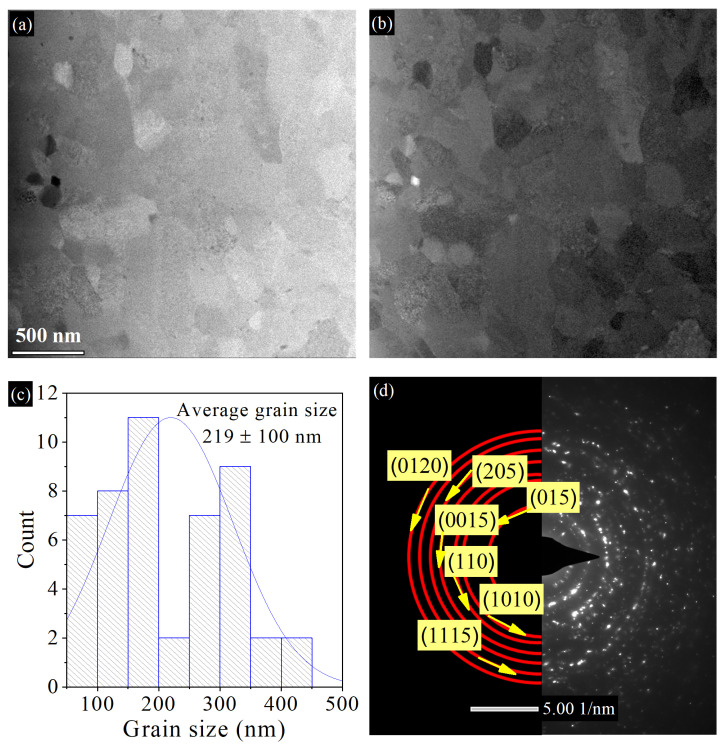
(**a**) Bright-field TEM image, (**b**) dark-field TEM image, (**c**) grain size distribution, and (**d**) diffraction pattern for HP Gr-Bi_2_Te_2.55_Se_0.45_ pellet at 350 °C and 1 GPa.

**Figure 3:**
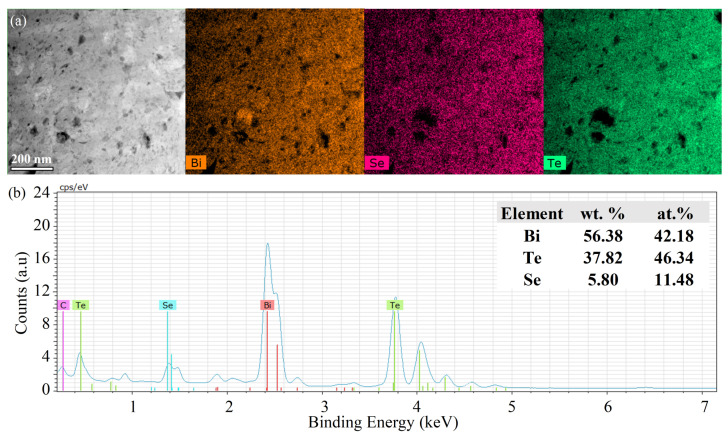
(**a**) HAADF-STEM image and the corresponding EDX elemental maps showing the elemental distributions of Bi, Te, and Se in the HP Gr-Bi_2_Te_2.55_Se_0.45_ pellet at 350 °C and 1 GPa. (**b**) The corresponding EDS spectrum and wt.% of each element.

Figure 2a shows the bright-field TEM image of the hot-pressed Gr-Bi_2_Te_2.55_Se_0.45_ nanocomposite at 350 °C and 1 GPa. It can be seen that the grains are equiaxed and randomly distributed within the structure. The average grain size was calculated from several dark-field TEM images to be 219 ± 100 nm with no grains above 446 nm (Figure 2b,c). On another note, the as-milled 0.05 wt.% Gr-Bi_2_Te_2.7_Se_0.3_ powders prepared by the same ball milling parameters have been reported by our group to have a grain size of ~20 nm [11,30]. Hence, the 350 T sample has experienced grain growth after HP, which is expected since the pressing temperature is near the reported grain growth temperature of the milled Bi_2_Te_2.7_Se_0.3_ alloy [30]. The electron diffraction patterns shown in Figure 2d reveal the atomic planes of the pseudohexagonal Bi_2_Te_2.55_Se_0.45_ nanograins, matching well with the XRD patterns. Figure 3a shows (HAADF) STEM imaging with EDX elemental mapping. It can be observed that (1) the distribution of Bi, Te, and Se is uniform throughout the sample, and (2) Se and Te are missing from some areas, supporting the evaporation of elements proposed in the XRD results due to the noticed shift in peaks to higher angles, as reported by [31]. The corresponding EDS of the mapped area is presented in Figure 3b. As noticed from at.% of the tested area, an apparent loss of about 4.7 at.% in Te is observed (originally 51 at.%), indicating noticeable evaporation of Te.

**Figure 4:**
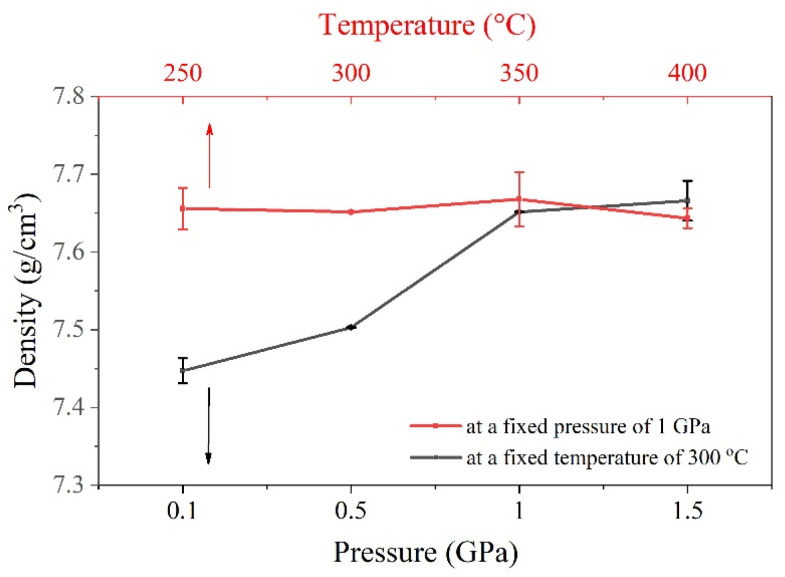
Density of Gr-Bi_2_Te_2.55_Se_0.45_ pellets at different HP conditions.

**Figure 5:**
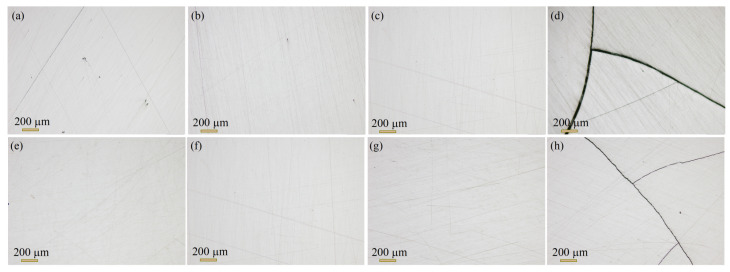
Optical micrographs of HP pellets: (**a**) 0.1 P, (**b**) 0.5 P, (**c**) 1 P, (**d**) 1.5 P, (**e**) 200 T, (**f**) 250 T, (**g**) 300 T, and (**h**) 400 T.

The density of HP samples at different temperatures and pressure conditions is presented in Figure 4. As presented, when the temperature was fixed at 300 °C (black curve), increasing the pressure raised the density from 7.44 ± 0.02 g/cm^3^ at 0.1 GPa to a maximum density of 7.67 ± 0.03 g/cm^3^ for the sample pressed at 1.5 GPa. This increment in density could be attributed to the higher pressing forces used, which led to better adhesion of the particles. Moreover, as seen in the micrographs of HP pellets in Figure 5, the surface of pellets pressed at 1 GPa or lower is found to be smooth and almost pore-free. However, at a high pressure of 1.5 GPa, HP pellets tend to form large cracks, making them quite fragile for further measurements and applications. It is worth noting that the density at 1 GPa and 1.5 GPa is almost the same, and further pressure increases lead to cracking. Hence, a pressure of 1 GPa was used as an optimized value to investigate the temperature.

Moreover, increasing the temperature while fixing the pressure at 1 GPa (red curve) has no significant effect on the density. This outcome is in disagreement with other reports. Yang et al. [32] prepared an n-type Bi_2_Te_2.85_Se_0.15_ alloy by ball milling and studied the effect of changing the HP temperature from 340 to 500 °C. The authors reported a remarkable increase in relative density, from 92.8 to 98.6%. The noticeable effect of temperature in their study may be attributed to the low pressure of 60 MPa and long holding times of 2, 4, and 5 h, giving more room and time for particle adhesion. In our study, high density (7.67 g/cm^3^) was obtained at a much shorter time and a lower temperature, indicating that adequately optimized pressing conditions could play an essential role in improving element migration and mass transfer, which boost densification during the HP process. It was also noticed here that the high temperature of 400 °C cracks the pellets (Figure 5). These results suggest that the Gr-Bi_2_Te_2.55_Se_0.45_ alloy can endure a maximum pressure and heat of about 1 GPa and 350 °C, respectively. According to the results in Figure 4, these conditions yield a maximum density of 7.67 g/cm^3^. 

**Figure 6:**
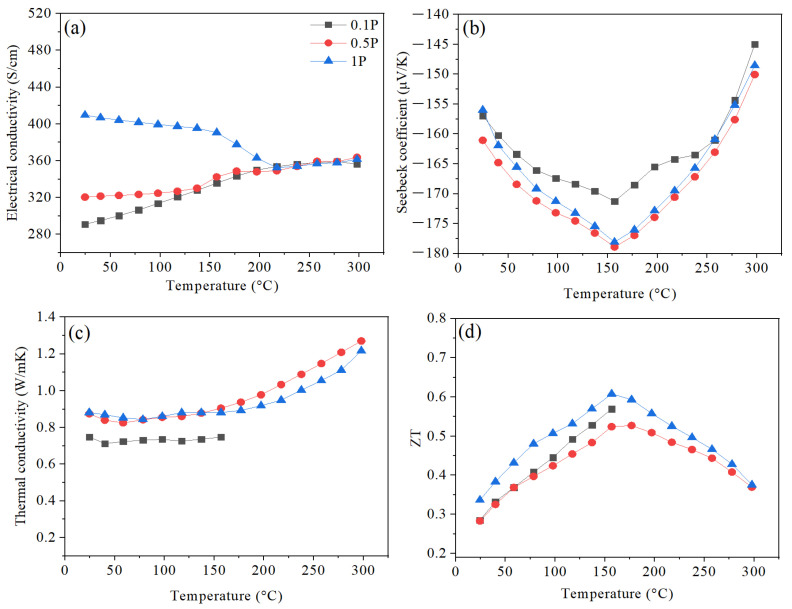
Temperature dependence of (**a**) electrical conductivity, (**b**) Seebeck coefficient, (**c**) thermal conductivity, and (**d**) ZT for HP pellets hot pressed at 0.1, 0.5, and 1 GPa.

Figure 6 presents changes in electrical conductivity (*σ*), Seebeck coefficient (S), total thermal conductivity (*κ*), and figure of merit for hot-pressed samples at different pressure values of 0.1, 0.5, and 1 GPa. The effect of HP pressure on electrical conductivity can be divided into two distinct phases (Figure 6a). Initially, from RT up to 200 °C, increasing the pressing raises the electrical conductivity trends significantly. Then, after 200 °C, the electrical conductivity of all samples reaches a comparable equilibrium value of ~360 S/cm. The electrical conductivity is mainly decided by the electrical band structure of the TE material and is defined as follows [33]:

(2) σ=neμ   

where *n* is the charge carrier concentration, *e* is the charge per carrier, and *µ* is charge carrier mobility. According to Equation (2), the electrical conductivity is directly proportional to the mobility and charge carrier concentration. Moreover, from Figure 4, higher pressure values increase the densification of pellets, as illustrated in Figure 4. This finding indicates better particle adhesion and less porosity with increasing the applied pressure, which reduces the carrier scattering and enhances the electrical conductivity. Hence, improved electrical conductivity with higher pressure mainly originates from the enhanced mobility of the charge carriers.

The three samples exhibit negative Seebeck coefficient values, as shown in Figure 6b, indicating that the major charge carriers are electrons. It can also be seen that increasing the applied pressure increases the Seebeck coefficient values. It can be noticed that initially, the absolute values of the Seebeck coefficient increase with temperature, reaching a maximum value of −157, −161, and −155 µV/K for 0.1 P, 0.5 P, and 1 P samples, respectively, at 160 °C (T_s,max_), then decrease again with increasing the temperature. Since the T_s,max_ of all samples is the same, changing the HP pressure does not affect the excitation temperature of the samples [5]. The degradation of the Seebeck coefficient can be explained by the excitation of minority charge carriers (holes), causing the bipolar effect. The Seebeck coefficient can be mainly represented using a simple model of electron transport [34]:

(3) $$S=\frac{8\;\pi^{2}k_{B}^{2}}{3\;e\;h^{2}}\;m^{*}T{\left(\frac{\pi }{3n}\right)}^{\frac{2}{3}}\;\;\;$$

where *k_B_* is the Boltzmann constant, *T* is the absolute temperature, *n* is the carrier concentration, *e* is the charge per carrier, m* is effective mass, and *h* is Planck’s constant. Contrary to the electrical conductivity, the Seebeck coefficient is inversely proportional to carrier concentration. Therefore, an increase in electrical conductivity often leads to a decrease in the Seebeck coefficient [1]. However, the electrical conductivity and Seebeck coefficient do not exhibit inverse coupling, supporting the idea that improved electrical conductivity resulted from improving carrier mobility rather than increasing the carrier concentration. 

The temperature-dependent total thermal conductivity (*κ*) is given in Figure 6c. The *κ* values of all samples show similar behavior as they increase with respect to temperature. It can be noted that the lowest pressure has the smallest *κ* value and the lowest density (Figure 4), indicating that porosity plays an essential role in scattering phonons and lowering the lattice thermal conductivity. The temperature dependence of ZT is shown in Figure 6d. It is clear that ZT for the highest pressure (1 GPa) outperforms the other samples over the entire temperature range studied. As discussed previously, an increase in pressure leads to an increase in density, carrier mobility, and absolute S values. The maximum ZT of 0.61 can be realized at around 160 °C in the 1 GPa sample, representing a 7% enhancement compared with the 0.5 GPa sample (ZT = 0.57). 

**Figure 7:**
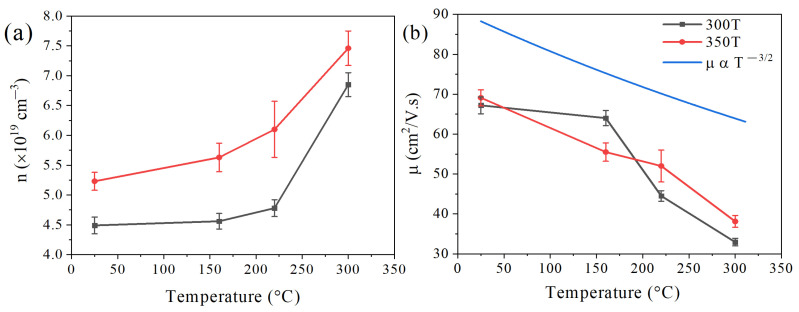
Temperature dependence of (**a**) charge carrier concentration and (**b**) Hall mobility for HP pellets at 300 T and 350 T.

Figure 7 shows the temperature dependence of charge carrier transport properties for HP pellets at 300 T and 350 T. It can be noticed that increasing the HP temperature from 300 °C to 350 °C has increased carrier concentration (n) through the entire temperature range studied (Figure 7a). In fact, it has been reported that the chemical composition greatly affects the concentration of charge carriers [35]; hence, the variation in the observed concentration may be attributed to the noticed fluctuation of composition due to Se and Te volatilization under different HP temperatures. The volatilization processes can be expressed as (i) the volatilization of *Te* atoms producing holes (*h*) [36,37]:

(4) $$Bi_{2}Te_{3}=2Bi_{Te}+2V_{Bi}+V_{Te}+\left(\frac{2}{3}\right)Te_{2\left(g\right)}+2h\;$$

where *Bi_Te_* is the occupation of *Bi* atoms into *Te* sites (anti-site defect); *V_Bi_* and *V_Te_* are vacancies of *Bi* and *Te* atoms, respectively; and (ii) the volatilization of *Se* atoms generating electrons is (*e*) [37,38]:

(5) $$3Bi_{2}Te_{3}=4Bi_{Bi}+2Bi_{se}+7V_{se}+2V_{Bi}^{1.5}+\left(\frac{9}{2}\right)Se_{2\left(g\right)}+2e\;$$

where *Bi_se_* is the occupation of *Bi* atoms into Se sites (anti-site defect) and *V_Se_* is a vacancy of the *Se* atom. Since *Se* has a lower energy of evaporation (37.70 kJ/mol) compared to *Te* (52.55 kJ/mol) [29], more *V_Se_* vacancies are expected to be formed compared to *V_Te_* in Bi_2_Te_2.55_Se_0.45_ alloys. Hence, many free electrons (carriers) will be produced, increasing the electrical conductivity of samples at higher HP temperatures. One additional process to consider is the interaction of the generated vacancies with anti-site defects, leading to a further increase in electrons [39,40]:

(6) $$2V_{Bi}+3V_{Te}+Bi_{Te}=V_{Bi}+Bi_{Bi}+4V_{Te}+6e\;\;$$

Therefore, higher HP temperatures lead to higher carrier concentrations and electrical conductivity values. 

Figure 7b shows the effect of increasing HP temperature on carrier mobility (µ). It can be noticed that increasing pressing temperature increases carrier mobility at RT. This can be attributed to the resulting grain growth and associated long effective mean-free path of carriers, which causes a reduction in the grain boundaries and scattering center [41,42]. Regardless of the HP temperature, the electrical mobility decreases with the ambient temperature, indicating that the dominant scattering mechanism is attributed to acoustic phonons. This mobility-temperature relationship possesses the power law of µα T^−3/2^ (see Figure 7b), which is consistent with what Cai et al. [43] have previously reported. It is worth noting that there are different scattering mechanisms that could take place during transport at different ambient temperatures. For example, ionized impurity scattering is more dominant at low temperatures in moderately doped semiconductors such as the samples in this study (T < 300 K). On the other hand, acoustic phonon scattering becomes more effective at higher temperatures (T > 300 K) [44]. 

**Figure 8:**
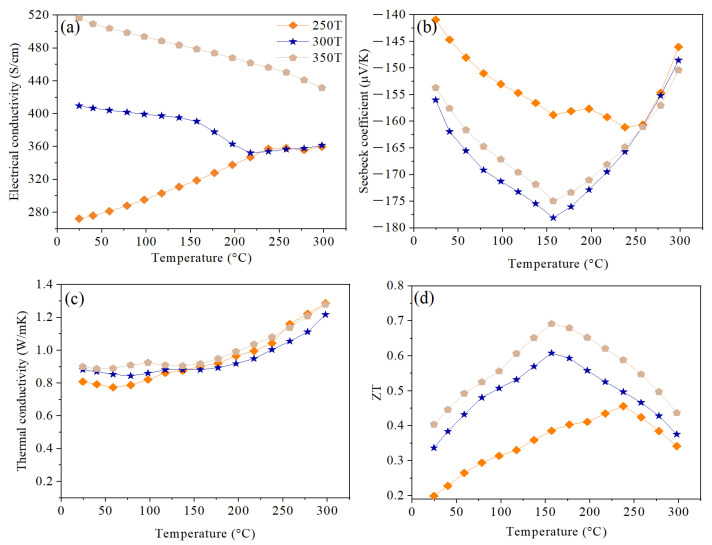
Temperature dependence of (**a**) electrical conductivity, (**b**) Seebeck coefficient, (**c**) thermal conductivity, and (**d**) ZT for HP pellets hot pressed at 250, 300, and 350 °C.

Figure 8a shows the temperature dependence of electrical conductivity for HP samples at different temperatures of 250, 300, and 350 °C. It can be noticed that a higher pressing temperature results in higher electrical conductivity values. It is well known that increasing the sintering temperatures helps reduce the internal energy presented in the form of defects generated during ball milling (such as a high dislocation density) [31]. This reduction in defects leads to less carrier scattering, thus higher mobility (Figure 7b) and conductivity. On another note, 250 T and 300 T samples exhibit similar electrical conductivity behavior as the samples discussed previously (0.1 P, 0.5 P, and 1 P), where the effect can be divided into two distinct phases around 200 °C (Figure 6a). However, this trend is not observed in 350 T. The trend change may be attributed to the grain growth of the Bi_2_Te_2.55_Se_0.45_ matrix, which is reported to occur near 350 °C [30]. An increase in grain size, accompanied by a smaller number of grain boundaries, is reported to reduce the scattering further, adding another factor to enhance the electrical conductivity. Furthermore, this enhancement in the electrical conductivity of the 350 T sample can be attributed to the increase in the carrier concentration compared to the 300 T sample (see Figure 7a). 

Figure 8b shows the Seebeck coefficient trends as a function of HP temperature. All samples exhibit n-type behavior since electrons are the main charge carriers, and higher pressing temperatures gave a higher magnitude of the Seebeck coefficient. It is interesting to note that changing the HP temperature changed the excitation temperature (T_s,max_) of the samples. A higher T_s,max_ of 240 °C was observed at lower pressing temperatures (250 °C), while samples pressed at 300 and 350 °C had a lower T_s,max_ of 160 °C. In fact, Femi et al. [45] reported that the amount of milling-induced defects and the generated density of interfaces/grain boundaries affect the band gap of BiTe alloys, hence causing a shift of the bipolar effect to a different temperature. The Goldsmid-Sharp bandgap (*E_g_*) was calculated using the following equation:

(7) $$E_{g}=2e\left|S_{max}\right|T_{max}\;$$

where *e* is the electron charge carrier, and the estimated values are shown in Table 2. It is observed that *E_g_* for the 250 T sample (0.165 eV) is larger than the rest (~0.15 eV). This can be explained by the higher density of grain boundaries and milling-induced defects in this sample since the HP temperature is low, causing strong selective filtering and an upshift in *S_max_* to a higher temperature. 

It is worth noting that even though the 300 T and 350 T samples have similar T_max_ and E_g_ values, the behavior of charge carrier transport properties is different. Figure 7b shows that the reduction rate of mobility for 350 T is quite consistent throughout the entire temperature range studied, unlike the 300 T sample. For 300 T, the mobility is somehow stable from RT up to T_max_ (160 °C), then the mobility drops sharply, implying that the excitation of minor charge carriers is the main reason for the deteriorating mobility in this sample. Moreover, from Figure 7a, it is clear that the carrier concentration is also stable before T_max_ (160 °C), then it increases exponentially. On the other hand, in the 350 T sample, the concentration of charge carriers starts to increase from RT, and then the rate rises further after T_max_. This implies that the number of broken bonds due to grain growth in the 350 T sample increases carrier concentrations and explains the drop in mobility at earlier temperatures.

The thermal conductivity behavior of the same samples is shown in Figure 8c. It is observed that as HP temperature increases, the thermal conductivity of the sample increases, and this can be attributed to two main reasons. First is the faster mobility of charge carriers due to larger grains and a lower density of the grain boundary, which increases the electronic part of thermal conductivity. Second is the lower concentration of milling-induced lattice defects, thus decreasing phonon scattering and increasing the lattice part of thermal conductivity. 

Figure 8d shows the figure-of-merit trends of HP samples at different temperatures. It is noticed that ZT for the highest temperature applied (350 °C) outperforms the other samples over the entire temperature range studied. As discussed, an increase in the temperature applied leads to a reduction in milling-induced defects, lower grain boundary density, and a higher Seebeck coefficient. The maximum ZT of 0.69 can be realized at around 160 °C in the 350 T sample, representing a 77% enhancement compared to the 250 T (ZT = 0.39). 

It is worth noting that varying HP temperatures have a greater effect on the thermoelectric properties of Gr-Bi_2_Te_2.55_Se_0.45_ nanocomposites than the applied pressure. In fact, several studies reported the effect of the sintering/HP temperature of mechanically alloyed n- and p-type Bi_2_Te_3_ powders on the thermoelectric properties [32,41,42,46,47]. Yang et al. [32] prepared n-type Bi_2_Te_2.85_Se_0.15_ using ball milling and HP techniques. They studied the effect of changing HP temperature and pressing duration while fixing the pressure at 60 MPa. An increase in both parameters (temperature and holding time) showed an increment in ZT first, then after a certain temperature/time, the ZT dropped again, implying optimum temperature and holding time parameters at a pressure of 60 MPa. Moreover, the highest ZT obtained for the best HP sample at 500 °C for 4 h is very low (around 1.7 × 10^−3^). Similar outcomes were observed with Fan et al. [46] when they prepared n-type Bi_2_Te_2.85_Se_0.15_ by mechanical alloying and plasma-activated sintering at various temperatures of 320, 350, 380, 410, and 440 °C, a holding time of 15 min, and a pressure of 30 MPa. Therefore, it is expected that there is an optimum temperature higher than 350 °C for the pressure value of 1 GPa used in our study.

The ease of producing Gr-Bi_2_Te_2.55_Se_0.45_ nanocomposites by ball milling and hot-pressing techniques makes them a promising candidate for developing sustainable thermoelectric devices for commercial applications. Optimizing the hot-pressing parameters, which are considered an important factor in dedicating the transport properties and thus improving the ZT to ~0.7, is a considerable step into commercializing these nanocomposites (see ZT comparison to other n-type materials prepared by ball milling followed by consolidation as presented in Table 3). The thermoelectric performance could be further maximized by considering (i) annealing the discs after hot pressing, as annealing can be beneficial for releasing unwanted stresses, thus boosting the electrical conductivity of the samples; (ii) testing the obtained optimized parameters on different n-type materials; or (iii) investigating other 2D nanofillers, such as MXene, to prepare the same Bi_2_Te_2.55_Se_0.45_ composite with optimized conditions. It is worth noting that there are a few sources of error that may have affected the interpretation of the obtained results, including (i) accurate measuring of the actual sample pressing temperature since the thermocouple is placed on the outer surface of the pressing die, and (ii) error in applying the exact amount of pressure needed, especially with small loads, since the pressure is added manually. Nevertheless, the obtained trends, supported by different characterizations performed, imply that the overall conclusions made in this work are quite credible and reasonable.

## 4. Conclusions

In summary, hot pressing of Gr-Bi_2_Te_2.55_Se_0.45_ nanocomposites was performed at different temperatures and pressure conditions in order to optimize the ZT. At first, various pressures of 0.1, 0.5, 1, and 1.5 GPa were considered at a fixed temperature of 300 °C and a holding time of 5 min. Analyzing the thermoelectric performance showed that increasing the pressure improves the densification of the samples and enhances the electrical transport properties and Seebeck coefficient, resulting in higher ZT values. Temperature optimization was then conducted at various temperatures of 250, 300, 350, and 400 °C, a fixed pressure of 1 GPa, and a holding time of 5 min. The results showed that increasing the pressing temperature leads to the evaporation of Se and Te constitutes and grain growth. This, in turn, causes an exponential increase in carrier concentration and improvements in electrical conductivity and the Seebeck coefficient. It is worth noting that varying the hot press temperature had more effect on the thermoelectric properties than varying the applied pressure. On another note, using high pressing parameters of 400 °C and 1.5 GPa produced large cracks and made the pellets fragile for any measurement or application. The maximum ZT of 0.69 (at 160 °C) was obtained for the sample pressed at the highest temperature of 350 °C, which is indeed an excellent and competitive value when compared with results reported for this n-type Bi_2_Te_2.55_Se_0.45_ composition.

## Figures and Tables

**Table 1:** Hot press parameters of the processed samples.

Step 1: Pressure optimization		
Fixed parameters	Variable parameter: pressure, GPa	Sample ID
T = 300 °C t = 5 min	0.1	0.1 P
	0.5	0.5 P
	1	1 P
	1.5	1.5 P
Step 2: Temperature optimization		
Fixed parameters	Variable parameter: temperature, °C	Sample ID
P = 1 GPa t = 5 min	250	250 T
	300	300 T (=1 P)
	350	350 T
	400	400 T

**Table 2:** Estimation of Goldsmid–Sharp bandgap for HP samples at different temperatures.

Hot Pressing Temperature (°C)	|Smax| µV/K	Tmax (°C)	Goldsmid–Sharp Bandgap (eV)
250	161.11	240	0.165
300	178.09	160	0.154
350	174.95	160	0.152

**Table 3:** ZT comparison with other n-type TE materials prepared via ball milling followed by consolidation.

Composition	ZT	Temperature °C	Reference
Bi_2_Te_3_	0.59	150	[26]
Bi_2_Te_3_	0.25	200	[48]
BiSe	0.27	250	[49]
Mg_2_Sn	0.62	350	[50]
Gr-Bi_2_Te_2.55_Se_0.45_	0.69	160	This work

## Data Availability

Data will be made available upon request.
